# Tacrolimus intrapatient variability and rejection are associated with inferior allograft outcomes after kidney transplantation

**DOI:** 10.3389/fneph.2025.1666191

**Published:** 2025-12-12

**Authors:** Maryam Javed, Aruna Sanghera, Azhar Ali Khan, Ria Nagpal, Katie Butler, Abigail Hobill, Alice Gage, Felix Karst, Amy Needleman, Mya Hmun, Nicola Thal, Graham Shirling, Ray Fernando, Gareth Jones, Mark Harber, Rhys D. R. Evans

**Affiliations:** 1 Department of Renal Medicine and Transplantation, Royal Free Hospital National Health Service (NHS) Foundation Trust, London, United Kingdom; 2 University College of London (UCL) Centre for Kidney and Bladder Health, Royal Free Hospital, London, United Kingdom; 3 Solid Organ Transplant Team, Anthony Nolan Research Centre, London, United Kingdom

**Keywords:** rejection, adherence, tacrolimus, outcomes, graft failure

## Abstract

**Introduction:**

Early kidney transplant failure has significant negative impact for individuals and healthcare systems. Contemporary data investigating early allograft failure are lacking. We undertook a retrospective observational cohort study of adult patients who underwent kidney transplantation at a single European centre.

**Methods:**

We determined causes of allograft failure between 1 and 5 years after transplant and explored clinical variables present at 1 year that predicted allograft loss.

**Results:**

591 patients (median age 50 years, 64.1% male, and 44% white) were included; 531 (89.8%) had graft survival and 60 (10.2%) had graft loss between 1- and 5-years. Rejection was the primary cause of graft failure in 24 (40%) cases and 54% had undetectable tacrolimus levels prior to failure event. Female sex, serum creatinine at 1 year, the occurrence of rejection, and undetectable tacrolimus levels were associated with increased odds of graft loss. In subsequent analysis of 787 patients alive with a functioning graft at 1 year, recipient age, serum creatinine, proteinuria, any rejection episode, and tacrolimus intrapatient variability (IPV) at 1 yearwere associated with an increased hazard of graft loss.

**Discussion:**

Hence, graft losses were predominantly alloimmune mediated, often associated with non-adherence, and were predicted by tacrolimus IPV at 1 year.

## Introduction

Kidney transplantation offers significant benefits over other forms of kidney replacement therapy, both in terms of quality and quantity of life. Advances in donor selection, histocompatibility testing, surgical techniques, and immunosuppression use have meant that short term outcomes in kidney transplantation have significantly improved over the last 30 years. Rejection within the first year occurred in up to 1 in 5 kidney transplant recipients in the United States (US) in 2000 (1), compared to current acute rejection rates of 5-7% (2). Patient survival and death censored graft survival at 1 year are 99% and 99% after living donation, and 96% and 95% after deceased donation, respectively, in the United Kingdom (UK) (3). These excellent outcomes are based on landmark clinical trials in kidney transplantation that were primarily designed to demonstrate improvements in transplant outcomes in the short term (4–8). These short-term outcomes are being achieved despite an increasingly medically complex recipient population, with many also at higher immunological risk.

Despite this increase in the number of patients alive with a functioning graft at 1 year after kidney transplantation, 30-40% of kidney transplants still fail within the first 10 years after transplant, with a graft attrition rate of 3% annually after the first post-transplant year (9, 10). This equates to nearly 1000 graft failures each year in the UK (10). Median graft survival is 11.2 years after kidney transplantation in the US (11), and, in contrast to early graft outcomes, there have only been modest improvements in longer term graft survival (12). The consequences of early graft loss may be devastating both at an individual and healthcare system level. Mortality is increased three-fold after graft loss compared to before graft loss (13), with previous meta-analyses demonstrating a 12% mortality rate in the first-year post dialysis initiation (14). A failed kidney transplant is a major contributor to Human Leucocyte Antigen (HLA) sensitisation (15), and two thirds of highly sensitised patients waitlisted in the UK have previously been transplanted (10). Graft failure is becoming an increasingly common reason for needing a kidney transplant, adding further demand to transplant programs that already lack the necessary organ supply (16, 17). Moreover, there are significant financial implications, with total incremental lifetime medical costs of graft failure estimated to be $1.3billion in the US (18). Hence, understanding why grafts fail and being able to identify patients at risk of early graft failure are important.

The greatest impact of kidney transplant failure is when this occurs relatively early in the post-transplant period. Graft failure between 1- and 5-years post kidney transplantation results in a significant loss of time with a functioning graft compared to median survival. There are limited recent data on the causes of graft loss at this timepoint, with one previous historical cohort highlighting alloimmune injury and non-adherence with medications as important contributors (19). In this study, we investigate the causes of allograft failure at 1–5 years post kidney transplantation in a contemporary cohort of transplant recipients. We also determine whether graft failures are predictable based on clinical variables present at the time of transplantation and at 1 year.

## Patients and methods

### Study design, setting, and participants

We undertook a single-centre, observational, cohort study of kidney transplant recipients who underwent transplantation at the Royal Free Hospital, London, UK. We included adult patients (aged >18 years) who underwent kidney alone transplantation between January 2012 and December 2019, and who were alive with a functioning graft at 1 year. For our main analysis, we categorised patients into two groups: patients with graft failure between 1- and 5-years (‘graft loss’ group) and patients with graft survival to 5 years post-transplantation (‘graft survival’ group). Patients who died with a functioning graft between 1- and 5-years, and patients with graft survival who were transferred out prior to 5 years follow-up, were excluded from this initial analysis.

We also undertook an analysis of the association between clinical variables present at 1 year and patient and allograft outcomes. For this subsequent analysis we included patients who were transplanted between January 2012 and March 2020 who were alive with a functioning graft at 1 year and who had clinical data (including creatinine and proteinuria) recorded and available for analysis at this timepoint. We included patients regardless of follow-up time after 1 year and included patients with all outcomes thereafter.

Royal Free Hospital provides a tertiary kidney transplant service to patients in North London and Hertfordshire, UK. It serves an ethnically diverse population and around 130 kidney alone transplants are undertaken each year. Most patients are followed up indefinitely by the transplanting centre; around one quarter of patients are transferred back to a referral hospital between 3 and 6 months after transplantation based on the location of where recipients live. The immunosuppression and prophylaxis protocols are outlined in **Supplementary Data Sheet 1**. All recipients except those with a compelling contraindication (e.g. allergy) undergo induction with an Interleukin 2-receptor antagonist (IL2-RA; Basiliximab). Recipients are maintained on tacrolimus and mycophenolate mofetil (MMF) thereafter, with 70% of patients managed steroid-free (20). We adopt a pre-emptive management strategy for CMV, and protocol biopsies are not performed.

### Variables, data sources and measurement

Data were documented prospectively within electronic health records and retrospectively analysed. Clinical variables from the time of transplantation related to the donor (age, sex, donor type and cause of death), and related to the recipient (age, sex, ethnicity, cause of end stage kidney disease, nature and duration of kidney replacement therapy prior to transplant, transplant number, mismatch at HLA-A, -B, and -DR loci, and levels of HLA sensitisation determined by the calculated reaction frequency [cRF]) were recorded. Post-transplant variables including graft function post operatively, clinical variables present at 1 year post-transplant (creatinine, proteinuria, tacrolimus intrapatient variability [IPV], and the development of CMV and BK viremia), and clinical variables present at any time up to 5 years post-transplant (T cell mediated rejection [TCMR], antibody mediated rejection [ABMR], undetectable tacrolimus levels, development of malignancy, and occurrence of cardiovascular events) were also documented. Predominant causes of graft loss were determined by a clinician. We used an online risk communication tool (https://wintoncentre.maths.cam.ac.uk/projects/communicating-risks-and-benefits-around-transplant-surgery/) to determine predicted graft survival at the time of transplantation based on donor and recipient characteristics. Tacrolimus was measured with liquid chromatography mass spectrometry using a Simadzu 8050 analyser. A 6-value calibration curve was determined using the Chromsystems 6PLUUS1 multilevel MassTox Immunosuppressants in Whole Blood calibrator set with the lower limit of quantification 2ng/ml. Tacrolimus IPV was determined at 1 year after kidney transplantation using the coefficient of variance (COV) incorporating the previous 10 tacrolimus trough concentrations, with COV defined as (standard deviation/mean) x 100 (21).

### Outcome measures

Clinical variables were compared between graft loss and graft survival groups. In patients with graft loss, we determined predicted graft survival at 1-, 3-, and 5-years after transplant, causes of graft failure, and patient outcomes after graft failure event. We investigated clinical variables associated with graft failure. Thereafter, we determined clinical variables present at 1-year post-transplantation that were associated with patient and graft survival in the wider cohort.

### Statistical methods

Data are reported as number and percentages for categorical variables, and mean and standard deviation (SD) or median and interquartile range (IQR) for numerical variables, depending on data distribution. Categorical variables were compared using the Fisher’s exact or Chi-squared test. Numerical variables were compared between two groups using the Mann–Whitney or an unpaired *t* test, and across greater than two groups with a one-way analysis of variance. Multivariable logistic regression was undertaken to determine clinical variables associated with graft loss at 1–5 years. Odds ratios (OR) and 95% confidence intervals (CIs) were determined for each variable. Demographic variables (recipients age, sex, and ethnicity), variables known to impact graft outcomes (type of transplant, HLA mismatch and levels of sensitisation), and variables not otherwise captured with a p-value of <0.05 in univariable analyses (pre-emptive transplant, delayed graft function, creatinine at 1 year, any rejection episodes, tacrolimus IPV, and undetectable tacrolimus levels) were included in the model. This analysis was undertaken in the initial cohort of patients with graft loss between 1- and 5-years and those with graft survival to 5 years. Multivariable cox regression analyses were undertaken to determine clinical variables associated with patient death, graft loss, and death-censored graft loss. Hazard ratios (HR) and 95% CIs were determined for each variable. Demographic variables (recipients age, sex, and ethnicity), variables known to impact graft outcomes (HLA mismatch and levels of sensitisation), and clinical variables recorded at 1 year (creatinine, proteinuria, tacrolimus IPV, and occurrence of rejection, CMV viraemia and BK viraemia at any time in the first year) were included in the models. We also explored a simpler model to determine variables associated with death censored graft loss incorporating creatinine and proteinuria at 1 year only, akin to the functional integrative Box (iBox) (22, 23). We did this with and without the inclusion of tacrolimus IPV and determined the concordance probability of each model using Harrell’s C-statistic. Analyses were performed using GraphPad Prism version 10 (www.graphpad.com). A *p*-value of ≤0.05 was considered statistically significant.

### Ethics statement

The study involved the retrospective analysis of routinely collected clinical data and, as such, was exempt from formal review board approval.

## Results

### Cohort description

**Figure 1:**
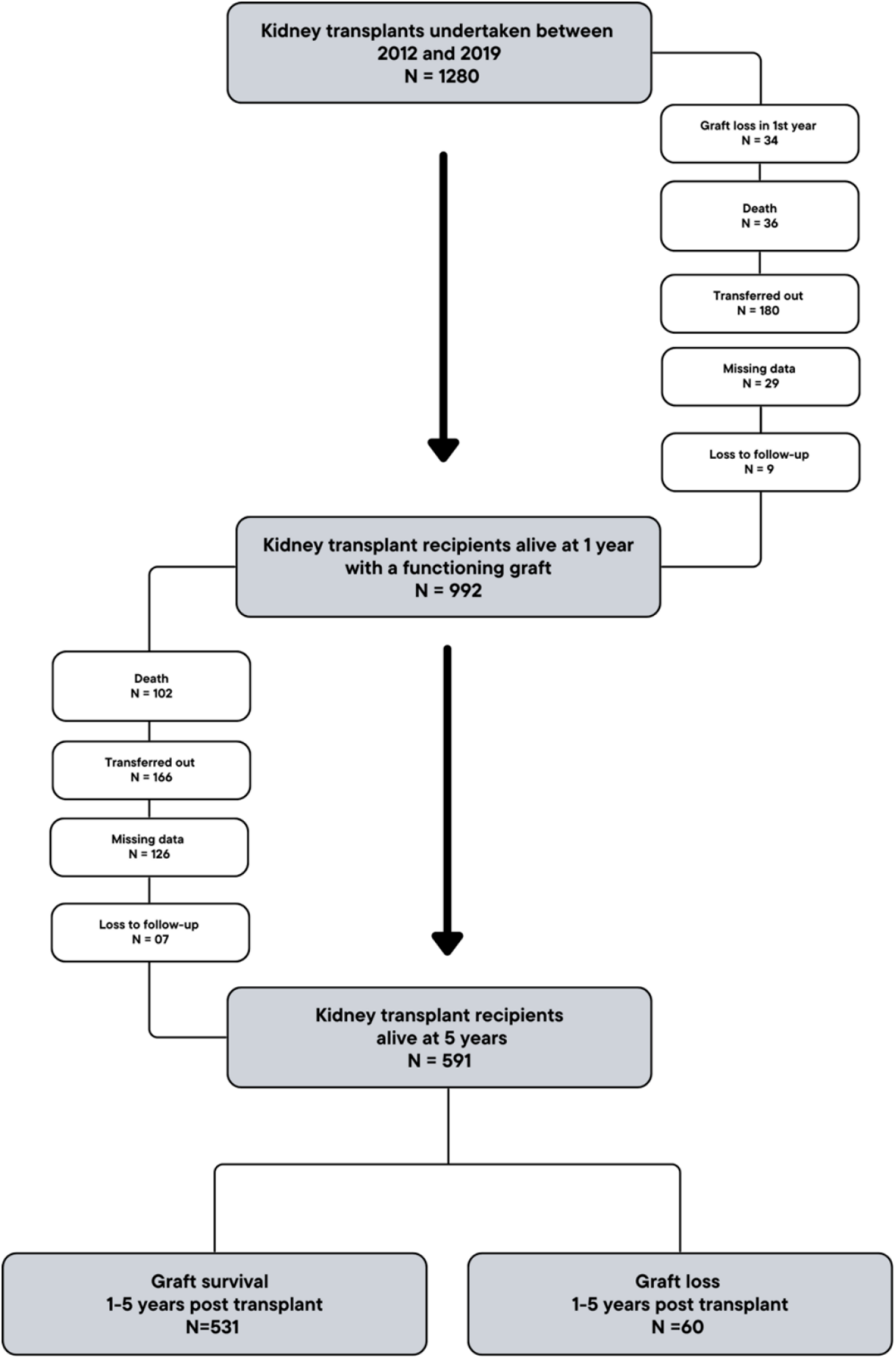
Cohort description.

1280 patients underwent kidney-alone transplantation between 2012 and 2019, and 992 were alive and under active follow-up with a functioning graft at 1-year. Of these, 591 patients were alive and completed follow-up to 5 years, and were included in the analysis (**Figure 1**). 531 (89.8%) patients had a functioning graft (‘graft survival’ group) and 60 (10.2%) patients had graft loss (‘graft loss’ group) during post-transplant years 1-5.

In the entire cohort, recipients were median age 50 (29-71) at the time of transplant, 379 (64.1%) were male, and 260 (44.0%) were of white ethnicity. 168 (28.4%) patients underwent living donor kidney transplant, and transplantation was pre-emptive in 156 (26.4%) cases. **Table 1** outlines clinical variables in donors and recipients in patients with graft survival to 5 years and in patients with graft loss between 1- and 5-years. Patients with graft loss less commonly underwent pre-emptive transplantation, had higher rates of delayed graft function, serum creatinine and tacrolimus IPV were higher at 1 year, and there were more rejection episodes in this group.

**Table 1:** Clinical variables in patients with graft survival to 5 years and patients with graft failure at 1–5 years post-transplant.

Clinical variable	Graft survival to 5 years post-transplant	Graft loss at 1–5 years post-transplant	P value
	N=531 (89.8%)	N=60 (10.1%)	
Donor Variables			
Donor type (n; %) -Live donor -Donor after brain death (DBD) -Donor after cardiac death (DCD)	156(29.3%) 231(43.5%) 144(27.1%)	12(20.0%) 26(43.3%) 22(36.6%)	0.1846
Cause of death in deceased donor (n; %) -Trauma -Cardiovascular -Other -Missing	50(9.4%) 295(55.5%) 24(4.5%) 162(30.5%)	5(8.33%) 39(65.0%) 4(6.6%) 12 (20%)	0.2946
Extended criteria donor (ECD) (n; %)	59(11.1%)	8(13.3%)	0.6662
Recipient variables			
Age at transplant (median; IQR)	50(38-59)	47(33-59)	0.7844
Gender (n; %) -Male -Female	345(64.9%) 186(35.0%)	34 (56.6%) 26 (43.3%)	0.2048
Ethnicity (n; %) -White -Asian -Black	234(44.0%) 157(29.5%) 140(26.3%)	26(43.3%) 12(20.0%) 22(36.6%)	0.1555
Cause of native kidney disease (n; %) Diabetes Non-Diabetes	104(19.5%) 427(80.4%)	14(23.3%) 46(76.6%)	0.4968
Body mass index (BMI) (median; IQR)	25.5(22.90-28.80)	25.8(22.43-29.98)	0.5644
Dialysis before transplant (n; %) -Haemodialysis -Peritoneal Dialysis -Pre-emptive Transplant	277(52.1%) 106(19.9%) 148(27.8%)	37 (61.6%) 15(25.0%) 8(13.3%)	**0.0394**
Time on dialysis (days) (Median; IQR)	815 (419-1534)	1221(470-1976)	0.0896
Allograft number (n; %) 1 2 3 4	145(89.6%) 13(8.0%) 3(1.8%) 1(0.6%)	53(88.3%) 7(11.6%) 0(0.0%) 0(0.0%)	0.6172
HLA Mismatch (n; %) 0 1 2 3 4 5 6	39 (7.3%) 27 (5.08%) 109 (20.5%) 197 (37.1%) 114 (21.4%) 30 (5.6%) 15 (2.8%)	04 (6.6%) 02 (3.3%) 13 (21.6%) 20 (33.3%) 16 (26.6%) 03 (5.0%) 02 (3.3%)	0.9699
Calculated reaction frequency at transplant (cRF) (n; %) <1% 1-84% 85-100%	306 (59.3) 170 (32.9) 40 (7.8)	40 (64.5) 15 (24.2) 7 (11.3)	0.2563
Post-transplant variables			
Graft function (n; %) -Primary Graft Function -Delayed Graft Function	410(78.2%) 114(21.7%)	33(55.0%) 27(45.0%)	**0.0002**
Induction agent (n; %) -Basiliximab -Campath -ATG -Unknown	520(97.9%) 4(0.7%) 6(1.1%) 1(0.2%)	58 (96.6%) 2 (3.3%) 0 (0.0%) 0 (0.0%)	0.2997
Creatinine at 1 year (umol/l) (median; IQR)	122(101-150)	182(134-245)	**<0.0001**
Intrapatient tacrolimus variability (Tac IPV) at 1 year (%) (median; IQR)	23.9(17.8-34.5)	30.53 (22.6-42.7)	**0.0006**
T-Cell Mediated Rejection at any time up to 5 years post-transplant (n; %)	45 (8.6%)	17 (28.3%)	**<0.0001**
Antibody Mediated Rejection at any time up to 5 years post-transplant (n; %)	11(2.0%)	7 (11.6%)	**0.0027**
Undetectable tacrolimus levels at any time up to 5 years post-transplant (n; %)	69 (12.9%)	32(53.3%)	**<0.0001**
CMV viremia (>3000 copies/ml) within first post-transplant year (n; %)	120 (22.8%)	11 (18.3%)	0.3885
BK viremia (any level) within first post-transplant year (n; %)	47(8.9%)	8(13.3%)	0.3441
Malignancy at any time up to 5 years post-transplant (n; %)	41(7.7%)	3(5.0%)	0.6068
Cardiovascular Event at any time up to 5 years post-transplant (n; %)	22(4.1%)	6(10.0%)	0.0540

Statistically significant results are highlighted in bold.

### Predicted graft outcomes at the time of transplantation in patients with early graft loss

**Figure 2:**
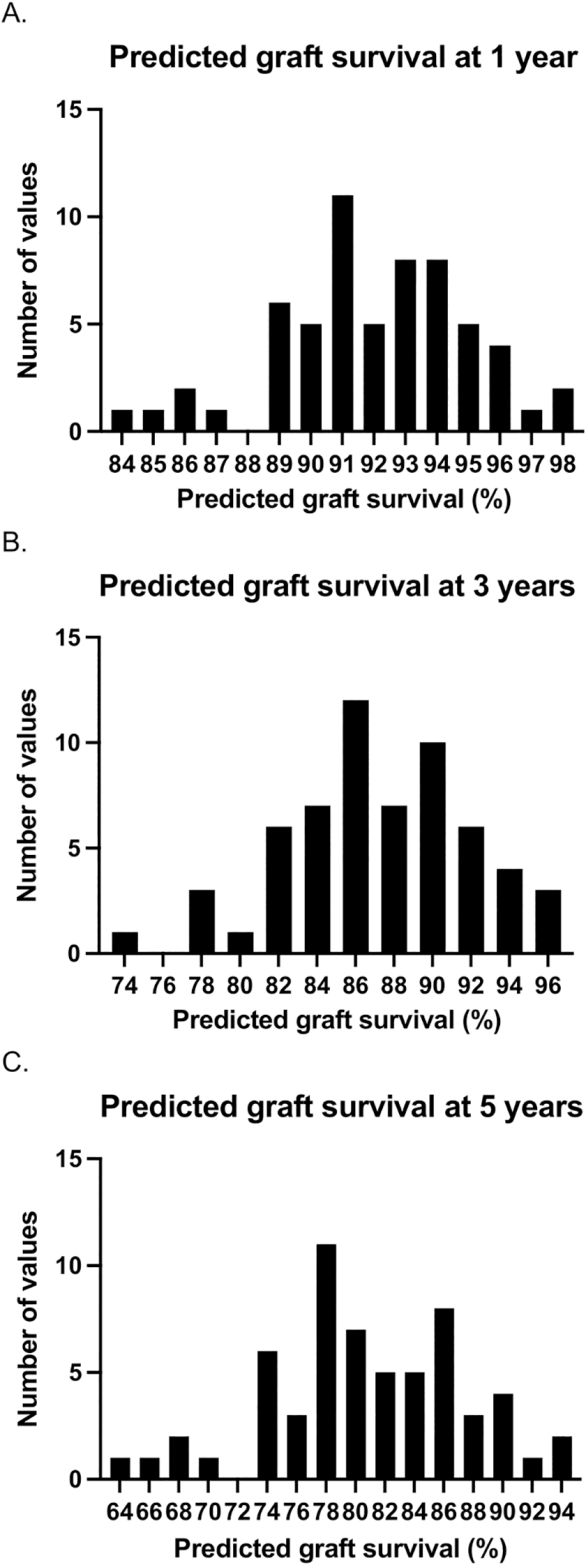
Predicted graft survival at 1- **(a)**, 3- **(b)**, and 5-years **(c)** post-transplant based on donor and recipient variables at the time of transplantation in those with early graft loss (between 1- and 5-years post-transplant). For each case, the prediction model determines the percentage of grafts that would be expected to be functioning at each follow-up timepoint. The histograms represent the number of cases with each graft survival prediction. For example, in 7 cases, it was predicted that 80% of grafts would survive to 5 years.

A risk calculator, incorporating donor and recipient variables at the time of transplantation, was used to determine the predicted graft outcomes in those patients with graft loss between 1- and 5-years. For each graft, based on the donor and recipient characteristics in that case, the prediction system provides the percentage of grafts expected to be functioning at 1-, 3-, and 5-years after transplant. **Figure 2** demonstrates the predicted graft survival at 1-, 3-, and 5-years post-transplant in patients with early graft loss using this model. The median predicted graft survival was 92% (90-94), 86.5% (84-90), and 80% (77-85) at 1-, 3-, and 5-years respectively (i.e. in those with early graft loss, grafts were predicted to be functioning at 5-years 80% of the time).

### Causes and outcomes of graft loss

**Table 2** outlines the primary causes of allograft loss in patients with graft failure between 1- and 5-years post-transplant. Alloimmune injury was the most common cause of graft failure, responsible for 24 (40%) cases. Of the 24 patients with graft loss due to rejection, 9 (37.5%) experienced an initial rejection event within the first post-transplant year (Acute TCMR = 5, Active ABMR = 2, mixed rejection =2) and 15 (62.5%) experienced their first rejection event at 1–5 years (Acute TCMR/mixed rejection = 8, Active ABMR = 1, chronic/chronic active ABMR = 6) (**Supplementary Data Sheet 2**).

**Table 2:** Causes of graft loss in years 1–5 post-transplant.

Causes of allograft loss	N = 60
Rejection: -TCMR -ABMR (including transplant glomerulopathy) -Mixed rejection	24 (40%) 13 07 04
Unresolved Acute Kidney Injury	12 (20%)
Infection: 1. BK nephropathy 2. Pyelonephritis	10 (16.6%) 03 07
Interstitial fibrosis and tubular atrophy (IFTA)	06 (10%)
Recurrent Glomerular Disease	07 (11.6%)
Unknown	1 (1.6%)

TCMR – T cell mediated rejection; ABMR – antibody mediated rejection.

To assess the impact of medication non-adherence on graft loss, we determined the rates of any undetectable tacrolimus levels prior to graft loss and at any time over 5 years of follow-up in patients with graft survival. Tacrolimus was undetectable in 68 (12.9%) patients with graft survival and 32 (53.3%) patients with graft loss (p < 0.0001). The first undetectable tacrolimus level occurred at median 1085 (344-1420) days prior to graft loss. In those with graft loss due to rejection, 13 (54.2%) patients had undetectable tacrolimus levels prior to the graft loss event.

Of the 8 patients with TCMR, 3 presented with severe kidney impairment requiring urgent dialysis. All patients with acute TCMR were treated with pulsed methylprednisolone, and antithymocyte globulin (ATG) was added in one case. Active ABMR was treated with pulsed methylprednisolone, plasma exchange, and intravenous immunoglobulin, whilst chronic and chronic active ABMR were managed with immunosuppression optimisation alongside non-immunomodulatory renoprotective measures. Most patients with graft loss remained on dialysis 1-year after graft failure (n=56, 93.3%; **Supplementary Data Sheet 3**). Only 20 (33.3%) patients were ultimately retransplanted over a median follow-up of 4.8 years.

### Multivariable analysis of clinical variables associated with graft loss at 1–5 years

A logistic regression analysis was undertaken to determine the variables associated with graft loss between 1- and 5-years post-transplant. Female sex (OR 2.35, 1.14-4.90), serum creatinine at 1 year (OR 1.02, 1.01-1.03), the occurrence of rejection (OR 2.83, 1.24-6.25), and an undetectable tacrolimus level (OR 7.71, 3.8-16.2) were associated with increased odds of graft loss (**Table 3**).

**Table 3:** Multivariable logistic regression analysis of clinical variables associated with graft loss at 1–5 years post-transplant.

Clinical variable	Odds ratio for graft loss at years 1-5	95% confidence interval
Age at Transplant	1.003	0.9780 to 1.028
Female Sex [reference = male]	**2.347**	**1.141 to 4.897**
Ethnicity [black; reference = white]	0.6926	0.2991 to 1.553
Ethnicity [Asian; reference = white]	1.005	0.4032 to 2.415
Pre-emptive transplant [reference = not pre-emptive]	0.5337	0.1857 to 1.403
DCD transplant [reference = live transplant]	0.8220	0.2531 to 2.677
DBD transplant [reference = live transplant]	0.8341	0.3097 to 2.306
HLA MM 1 [reference = HLA MM 0]	0.9370	0.1461 to 5.559
HLA MM 2 [reference = HLA MM 0]	0.3873	0.09433 to 1.772
HLA MM 3 [reference = HLA MM 0]	0.5349	0.1456 to 2.292
HLA MM 4 [reference = HLA MM 0]	0.7966	0.2045 to 3.591
HLA MM 5 [reference = HLA MM 0]	0.5778	0.07595 to 3.909
HLA MM 6 [reference = HLA MM 0]	0.2509	0.01045 to 2.538
cRF 1-84% [reference cRF = 0%]	0.6667	0.2928 to 1.448
cRF 85-100% [reference cRF = 0%]	0.5331	0.1305 to 1.834
Delayed graft function [reference = primary graft function]	1.512	0.6448 to 3.529
Creatinine at 1 year (umol/l)	**1.019**	**1.013 to 1.025**
Tacrolimus IPV at 1 year	1.001	0.9908 to 1.004
Rejection episode at any time 0–5 years [reference = no rejection]	**2.803**	**1.235 to 6.245**
Any undetectable tacrolimus level [reference = no undetectable tacrolimus level]	**7.705**	**3.760 to 16.20**

Statistically significant results are highlighted in bold.

Given the unexpected finding of the increased odds of graft loss in females in multivariable analyses, we explored reasons for this in more detail. Of 220 females included in the cohort, 26 (12.3%) suffered graft loss, compared to 34 (9.0%) of 379 males included. Rejection was the cause of graft loss in 11 (42.3%) females and 13 (38.2%) males; graft pyelonephritis was the cause of graft loss in 5 (19.2%) and 2 (5.8%) females and males respectively (p=0.29; **Supplementary Data Sheet 4**). Tacrolimus was undetectable prior to graft loss 17 (65.4%) females and 15 (44.1%) males (p=0.12).

### Predicting graft outcomes using clinical variables at 1 year

A cox regression analysis was undertaken to determine clinical variables at 1 year that were associated with patient and graft outcomes. For this analysis, we included all patients alive with a functioning graft at 1 year with relevant clinical data regardless of patient or allograft outcome thereafter. A total of 787 patients were included in this analysis; the clinical characteristics of this cohort are outlined in **Supplementary Data Sheet 5**. Patients were followed up for a median 2573 (1916-3333) days; 106 (13.5%) patients died, and there were 61 (7.8%) graft failures over this timeframe.

**Figure 3:**
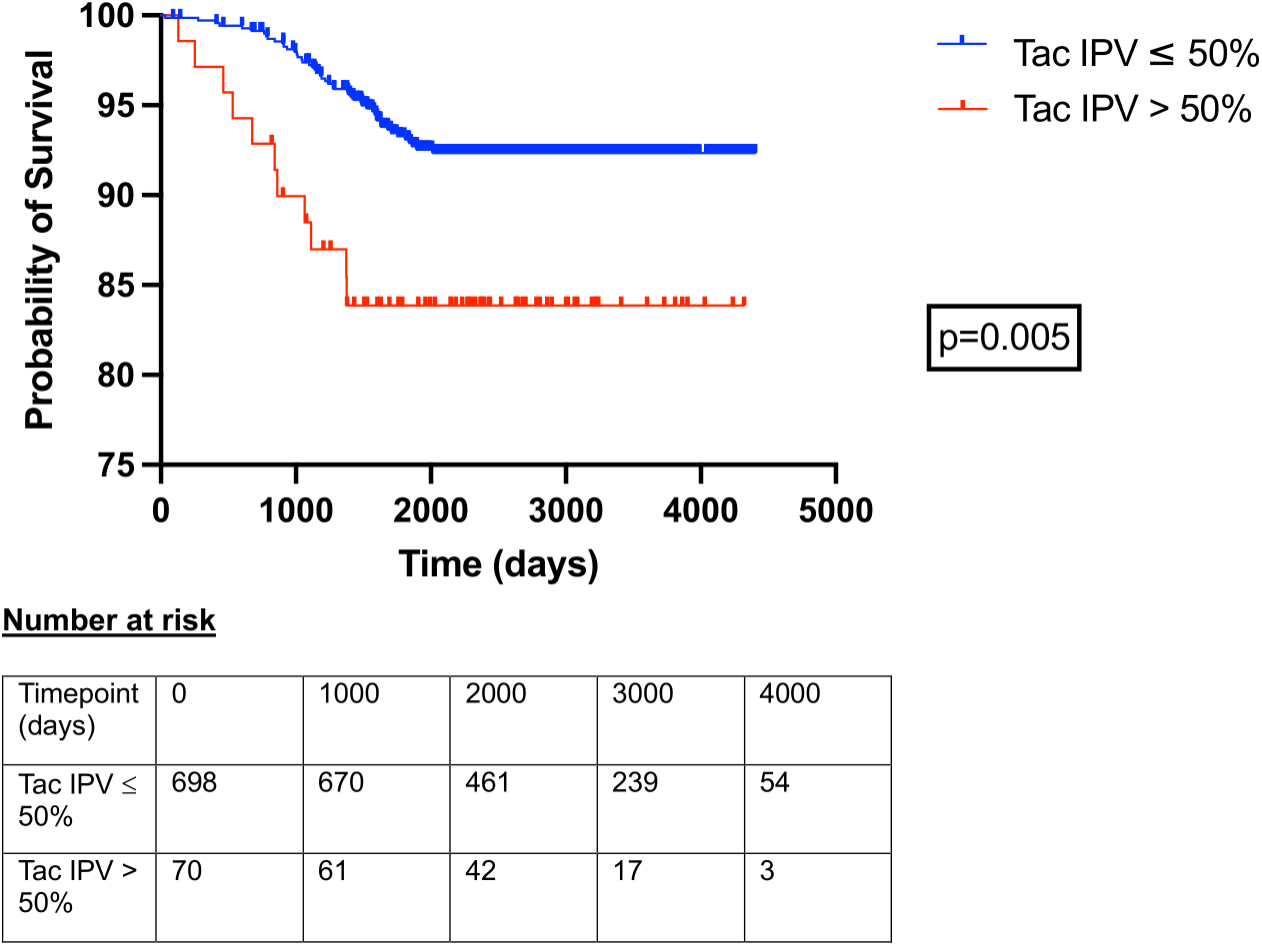
Death censored allograft survival in patients with tacrolimus IPV > 50% and ≤ 50% at 1 year post kidney transplant. Time 0 represents 1-year post-transplant. Curves compared with log-rank test.

Clinical variables associated with patient mortality, graft loss (graft failure or death with functioning graft), and death censored graft loss are outlined in **Table 4**. Tacrolimus IPV was included as a continuous variable; the same analyses with tacrolimus IPV included as a categorical variable are outlined in **Supplementary Data Sheet 6**. Recipient age (HR 1.035, 1.020-1.051), serum creatinine at 1 year (HR 1.006, 1.004-1.008), proteinuria at 1 year (HR 1.004, 1.002-1.005), any rejection episode within the first year (HR 3.074, 1.791-5.067), and tacrolimus IPV (HR 1.011, 1.004-1.017) were associated with an increased hazard of graft loss (**Table 4**). Female sex (HR 2.262, 1.218-4.187), serum creatinine at 1 year (HR 1.009, 1.006-1.011), proteinuria at 1 year (HR 1.004, 1.002-1.005), any rejection episode within the first year (HR 4.334, 2.011-8.768) and tacrolimus IPV (HR 1.011, 1.000-1.021) were associated with an increased hazard of death censored graft loss (**Table 4**). Hazard ratios for death censored graft loss in models incorporating only creatinine and proteinuria at 1 year, with and without tacrolimus IPV, are outlined in **Supplementary Data Sheet 7**. Harrell’s C-statistics were 0.816 (0.767-0.865) and 0.819 (0.767-0.871) in models with and without tacrolimus IPV respectively. Death-censored graft survival stratified by tacrolimus IPV at 1 year is shown in **Figure 3**.

**Table 4:** Cox regression analyses of 1-year clinical variables associated with patient mortality, graft loss (graft failure or death with functioning graft), and death-censored graft loss.

Clinical variable	Patient mortality		Graft loss		Death-censored graft loss	
Harrell’s C-Statistic (concordance probability) and 95% confidence interval	0.80 (0.76-0.84)		0.76 (0.72-0.80)		0.82 (0.77-0.88)	
	Hazard ratio	95% Confidence interval	Hazard ratio	95% Confidence interval	Hazard ratio	95% Confidence interval
Recipient age at transplantation	**1.071**	**1.050 to 1.094**	**1.035**	**1.020 to 1.051**	1.003	0.9812 to 1.025
Female Sex [reference = male]	0.8766	0.5237 to 1.434	1.223	0.8154 to 1.813	**2.262**	**1.218 to 4.187**
Ethnicity [black; reference = white]	0.8878	0.5028 to 1.523	0.6606	0.4037 to 1.051	0.5616	0.2345 to 1.233
Ethnicity [Asian; reference = white]	1.116	0.6504 to 1.880	0.8751	0.5679 to 1.333	0.8805	0.4582 to 1.661
Donor Age	**1.027**	**1.008 to 1.046**	1.009	0.9947 to 1.024	0.9818	0.9614 to 1.003
CRF at baseline	1.003	0.9947 to 1.010	0.9999	0.9938 to 1.006	0.9951	0.9857 to 1.004
HLA MM1 [reference = HLA MM 0]	0.3255	0.04670 to 1.460	0.6430	0.1398 to 2.248	1.337	0.1712 to 8.394
HLA MM 2 [reference = HLA MM 0]	0.5221	0.2084 to 1.489	0.9763	0.4598 to 2.322	1.087	0.3265 to 4.933
HLA MM 3 [reference = HLA MM 0]	0.6325	0.2656 to 1.758	0.8396	0.4029 to 1.977	1.157	0.3705 to 5.107
HLA MM 4 [reference = HLA MM 0]	0.5134	0.2098 to 1.450	0.6645	0.3048 to 1.611	1.119	0.3307 to 5.139
HLA MM 5 [reference = HLA MM 0]	0.6697	0.2431 to 2.026	0.7200	0.2892 to 1.895	0.6241	0.1045 to 3.646
HLA MM 6 [reference = HLA MM 0]	0.6783	0.1671 to 2.482	0.9349	0.2748 to 2.897	0.9696	0.04649 to 8.097
Creatinine (umol/l) at 1 year	1.002	0.9991 to 1.005	**1.006**	**1.004 to 1.008**	**1.009**	**1.006 to 1.011**
Proteinuria (mg/mmol) at 1 year	1.002	0.9996 to 1.003	**1.003**	**1.002 to 1.005**	**1.004**	**1.002 to 1.005**
Rejection episode at any time in 1^st^ year [reference = no rejection]	**2.359**	**1.164 to 4.477**	**3.074**	**1.791 to 5.067**	**4.334**	**2.011 to 8.768**
CMV viremia >3000 copies/ml at 1 year [Reference CMV negative]	1.097	0.6642 to 1.759	0.8573	0.5558 to 1.291	0.5833	0.2634 to 1.178
BK viremia any level at 1 year [Reference BKV negative]	1.372	0.5383 to 2.993	1.810	0.9548 to 3.160	1.900	0.7096 to 4.261
Tacrolimus IPV at 1 year	1.009	0.9987 to 1.018	**1.011**	**1.004 to 1.017**	**1.011**	**1.000 to 1.021**

Hazard ratios and 95% confidence intervals are provided for each variable included within the model. Tacrolimus IPV is included as a continuous variable. Statistically significant results are highlighted in bold.

## Discussion

### Key findings

Early graft failure after kidney transplantation causes significant harm to individuals and healthcare systems. Given the relative lack of contemporary data investigating early graft failure, we undertook a retrospective, observational, cohort study to determine causes of graft failure at 1–5 years after transplantation at a single centre in the UK. We assessed modifiable and non-modifiable clinical variables associated with graft loss. We compared 531 kidney transplant recipients with graft survival to 5 years and 60 patients with graft loss during post-transplant years 1–5 in an ethnically diverse population. Patients with graft loss had higher serum creatinine, more rejection episodes, and higher tacrolimus IPV at 1 year. Graft losses were not expected based on donor and recipient characteristics present at the time of transplantation. Rejection was the most common cause of graft failure and undetectable tacrolimus levels were identified in over a half of patients in the graft loss group. Female sex, creatinine at 1 year, and undetectable tacrolimus levels were associated with graft loss in multivariable analyses. In the wider cohort incorporating all patients alive with a functioning graft at 1-year, recipient age, creatinine and proteinuria, any rejection episode, and a higher tacrolimus IPV at 1 year were associated with graft failure.

### Interpretation

Advances in transplant medicine have significantly improved short-term outcomes after kidney transplantation, and the proportion of recipients alive with a functioning graft at 1-year has increased. Surgical complications have replaced rejection as the predominant cause of graft failure within the first post-transplant year (9, 24). However, there has been much less progress in combatting graft attrition thereafter, with graft failures between 1- and 5-years post-transplant having significant negative impact. Previous investigation has shown that most graft failures have an identifiable cause (25), and historical cohorts (summarised in **Supplementary Data Sheet 8**) demonstrate that rejection is the most common cause at 1–5 years (19, 24, 26). For example, in a US cohort of patients transplanted in the 1990s, 60% of death-censored graft losses between 1- and 5-years post-transplant were due to rejection, with 18% in the setting of patients discontinuing medications. In a European cohort transplanted between 1995 and 2005, rejection accounted for 82% of graft losses between 1- and 5-years in patients 18–39 years and 60% of graft losses in those >55 years (26). In this study there was an equal split between TCMR and ABMR as the type of rejection, with ABMR becoming more common beyond 5 years, whilst more recent European data highlight a greater contribution of ABMR at the 1–5 year timepoint (24). Our results from a contemporary kidney transplant cohort demonstrate little change from these historical findings. Rejection continues to be the predominant reason for graft failure between 1- and 5-years, responsible for 40% of cases in our cohort, with both cell and antibody mediated mechanisms involved. Non-adherence contributed to a large proportion of cases. This is consistent with a more recent cohort from the US (27), and our findings reinforce ongoing missed opportunities in the management of kidney transplant recipients within the first 5 years.

Our study also supports the concept that events beyond the immediate post operative period play an important role in graft failure between 1- and 5-years post-transplant (9). Suboptimal immunosuppression, which most often occurs in the setting of non-adherence to medications, is a potentially modifiable risk factor that leads to a rejection event and subsequent early graft loss. Non-adherence may be intentional or unintentional, and it remains a common problem within the transplant community. Unintentional non-adherence, for example, may occur in up to two thirds of recipients, with few, if any, proven strategies for its management (28, 29). Whilst there is no accepted or proven method to detect it, non-adherence is suggested by undetectable immunosuppression levels and by increases in tacrolimus IPV, and these were the features used to define non-adherence in this study. However, such features represent the worst-case scenario of non-adherence, and patients who intermittently but consistently miss individual doses of immunosuppression may not be identified with these methods. Missing immunosuppression in this manner can be impactful, and more accurate methods to identify non-adherence have been proposed. These include pill counting, electronic monitoring systems, and wireless observed therapies (28). These strategies weren’t possible given the retrospective nature of the study.

Conceptually, tacrolimus IPV can be considered as the fluctuation in whole blood tacrolimus concentration over a period of time (30). There is some variation in how and when it is calculated, but most studies assess its impact when determined based on tacrolimus levels measured between 3- and 12-months after transplant. Previously, higher tacrolimus IPV has been associated with inferior graft survival (21, 31–34), allograft rejection (35–38), the development of *de novo* donor specific antibodies (DSAs) (39), calcineurin inhibitor toxicity (40), and worse outcomes in patients with chronic active ABMR (41). We demonstrated that tacrolimus IPV was associated graft failure in both univariable and multivariable analyses and hence our findings add weight to its importance as a biomarker associated with adverse outcomes. In doing so, we demonstrate that alterations in patient behaviour that impact kidney transplant outcomes are already evident by 1-year post-transplantation and enhanced efforts focussed on identifying and addressing these behaviours are an essential part of making kidney transplants last longer.

Identifying patients at high risk of graft failure is an important step to enable novel management strategies to be initiated aimed at improving outcomes in this group. Graft survival prediction systems have recently been developed, with IBox the most well studied (22). Ibox uses clinical variables within a multivariable model to determine risk of allograft failure, and it has been validated in international cohorts and used in clinical trials (22, 23, 42). It performs best when undertaken at 1-year post kidney transplant and, with time, it has been simplified such that its latest iteration includes creatinine and proteinuria only (the ‘functional IBox’) (43–45). However, one major limitation of IBox is that the variables included within its model are largely non-modifiable. In our analysis, we confirmed that creatinine and proteinuria at 1 year associate with adverse graft outcomes, as has been shown in IBox, but we also explored modifiable variables such as tacrolimus IPV. We demonstrate that tacrolimus IPV is associated with worse graft survival, even when adjusting for creatinine and proteinuria at 1 year. Moreover, we highlight a subtle increase in the concordance probability of the prediction model when tacrolimus IPV is included compared to creatinine and proteinuria alone. Previous studies have shown that interventions can improve tacrolimus IPV, either through changing tacrolimus formulation or through behavioural methods (46–48). Moreover, improving tacrolimus IPV has been shown to improve graft survival (49). Our data support focussing on patients with increased tacrolimus IPV at 1 year (e.g. with enhanced use of novel biomarkers (50)), and we advocate for the development of novel strategies that reduce tacrolimus IPV as we anticipate these will lead to less rejection at 1–5 years and hence decrease early allograft loss.

### Limitations

In this study, we provide unique data on the causes of early allograft loss and variables associated with this loss in a large, contemporary cohort of kidney transplant recipients from Europe. Causes of graft failure were clinician determined and there may have been individual variation in the approach. We provided the predominant cause of graft failure but accept that this is often multifactorial. A diagnosis of rejection was based on biopsy findings, but comprehensive Banff scoring was not always available and molecular analyses of biopsies were not performed (51). Our centre does not undertake protocol biopsies and hence the contribution of alloimmune-mediated injury to graft failure may have been underestimated. Moreover, we could not include histological scores in our multivariable models. We lacked complete data on donor specific antibodies, and hence this variable was also excluded from the models. We also lacked data on the contribution of autoimmune diseases as the cause of native kidney disease, some of which may be associated with increased graft failure. Tacrolimus IPV was determined at 1 year, but there was no time restriction over which this calculation was made, and the tacrolimus levels included within the calculation may have been undertaken at any time prior to this timepoint. We collected data on undetectable tacrolimus levels in addition to IPV but didn’t include other measures of non-adherence given the retrospective nature of the study. Moreover, investigating if other factors unrelated to adherence (e.g. change in medications, acute illness) may have impacted IPV was beyond the scope of this project. We highlighted some variables, such as female sex, that were associated with worse graft outcomes, but the reasons underlying these findings were not answered by this study. We anticipate our findings are generalisable to many healthcare systems, albeit the ethnic diversity of the cohort, alongside our unique immunosuppressive protocol of using Basiliximab induction and a steroid free maintenance regimen in most patients, may mean it is not generalisable to all settings.

## Conclusion

In summary, rejection remains the most common cause of early graft failure between 1- and 5-years after kidney transplantation. These graft failures were not expected based on donor and recipient characteristics at the time of transplantation but were predictable based on serum creatinine, proteinuria, and tacrolimus IPV at 1 year. Identifying high-risk patients at 1-year post-transplant and initiating management strategies to improve adherence and reduce tacrolimus IPV may prevent early allograft loss.

## Data Availability

The raw data supporting the conclusions of this article will be made available by the authors, without undue reservation.
