# Supplementary material for: Tacrolimus intrapatient variability and rejection are associated with inferior allograft outcomes after kidney transplantation
Source: Front Nephrol. 2025 Dec 12;5:1666191. doi: 10.3389/fneph.2025.1666191 (PMC12740892; doi:10.3389/fneph.2025.1666191)
Supplement: Supplementary file 1 [file DataSheet1.docx]

**Supplementary 1:** Immunosuppression and infection prophylaxis protocol at the Royal Free Hospital, UK

| *Induction immunosuppression* | Basiliximab, 20mg administered intravenously on the day of transplant, repeated on postoperative day 4.  Intravenous methylprednisolone 500mg at induction, followed by 40mg methylprednisolone daily for 3 days, prednisolone 20mg orally for 1 week. Prednisolone is stopped on day 10 unless patients were on prednisolone prior to transplant, had rejection in the first 10 days or had a primary cause of end stage kidney disease of lupus or vasculitis, where prednisolone is continued at 5mg daily thereafter. |
| --- | --- |
| *Maintenance immunosuppression* | Tacrolimus, started 5 days prior to living donor kidney transplant and on the day of deceased donor kidney transplant targeting trough concentrations of 8-12ng/ml within the first 3 months, 6-8ng/ml from 3-12 months, and 5-7ng/ml thereafter.  Mycophenolate Mofetil (MMF) administered at 2g/day in month 1, 1.5g/day in months 1-12, and 1g/day thereafter. |
| *PCP prophylaxis* | Co-trimoxazole 480mg daily for 3 months |
| *TB prophylaxis* | Isoniazid 300mg daily for 9 months in patients at increased risk of latent tuberculosis (TB) |
| *CMV prophylaxis* | Not given. Valaciclovir is given for 1 month in recipients who are HSV IgG negative. Treatment for CMV is initiated at any level of viremia in seronegative recipients and at 2190 IU/ml in seropositive recipients. |

**Supplementary 2**: Characterisation of rejection in patients with allograft loss due to rejection in years 1-5.


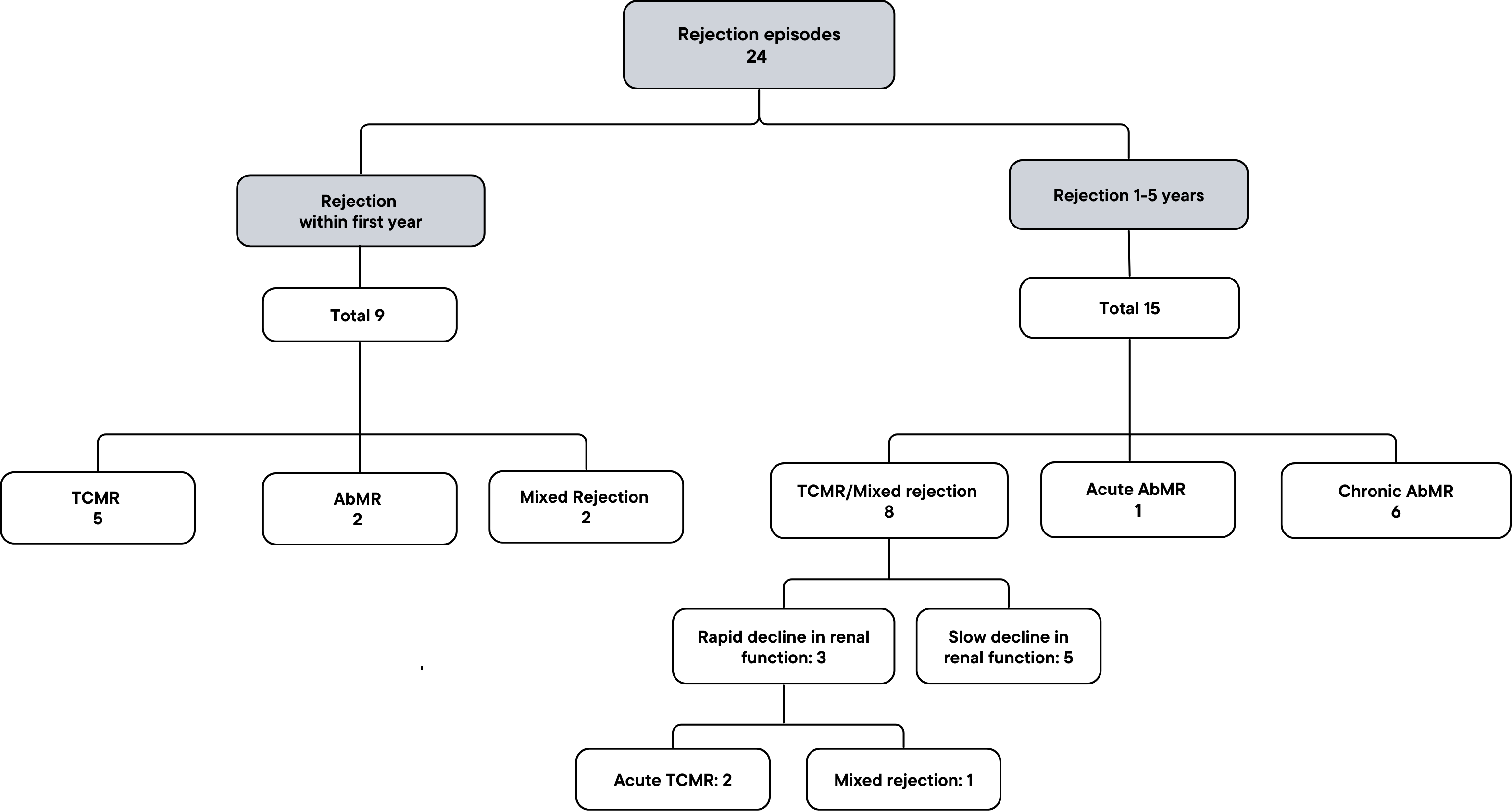


**Supplementary 3:** Causes of graft loss in males and females.

| **Causes of allograft loss** | **Female**  **N=26** | **Male**  **N=34** |
| --- | --- | --- |
| **Rejection:**  -TCMR  -ABMR (including transplant glomerulopathy)  -Mixed rejection | 11 (42.3%)  6  4  1 | 13 (38.24%)  7  3  3 |
| **Unresolved Acute Kidney Injury** | 4 (15.3%) | 8 23.5%) |
| **Infection:**  1. BK nephropathy  2. Pyelonephritis | 6  1 (3.8%)  5 (19.2%) | 4  2 (5.8%)  2 (5.8%) |
| **Interstitial fibrosis and tubular atrophy (IFTA)** | 4 (15.3%) | 2 (5.8%) |
| **Recurrent Glomerular Disease** | 1 (3.8%) | 6 (17.6%) |
| **Unknown** | 0 (0.0%) | 1 (2.9%) |

**Supplementary 4**: Patient outcomes in those with graft failure 1-5 years

|  | 1-year post graft loss | Final Outcome  (Median follow-up time 4.8 years) |
| --- | --- | --- |
| Haemodialysis | 51 (85%) | 22 (36.6%) |
| Peritoneal Dialysis | 05 (8.3%) | - |
| Transplantation | 02 (3.3%) | 20 (33.3%) |
| Transferred out | 01 (1.6%) | 12 (20%) |
| Died | 01 (1.6%) | 06 (10%) |

**Supplementary 5**: Clinical characteristics of patients alive at 1 year with a functioning graft included in the cox regression analysis of patient and allograft survival (n=787)

| **Recipient variables** | |
| --- | --- |
| Age at transplant (median; IQR) | 51 (39-60) |
| Gender (n; %)  -Male  -Female | 507 (64.4)  280 (35.5) |
| Ethnicity (n; %)  -White  -Asian  -Black | 362 (46.0)  216 (27.4)  209 (26.5) |
| Calculated reaction frequency (cRF) at transplant (n; %)  <1 %  1-84 %  85-100 % | 483 (61.4)  238 (30.2)  66 (83.9) |
| **Donor Variables** | |
| Donor age | 51 (40-60) |
| HLA Mismatch (n; %)  0  1  2  3  4  5  6 | 56 (7.1)  37 (4.3)  160 (20.3)  276 (35.0)  170 (21.6)  61 (7.7)  27 (3.4) |
| **Post-transplant variables** | |
| Creatinine at 1 year (umol/l) (median; IQR) | 127 (104-162) |
| Proteinuria at 1 year (mg/mmol)  (Median; IQR) | 17 (10-38) |
| Intrapatient tacrolimus variability (Tac IPV) at 1 year (%) (median; IQR) | 24.8 (18.1- 34.8) |
| Intrapatient tacrolimus variability (Tac IPV) at 1 year (n; %)  >50%  <50% | 70 (9)  701 (91) |
| Rejection within 1-year post-transplant (n; %) | 66 (8.4) |
| CMV viremia (>3000 copies/ml) within first post-transplant year (n; %) | 176 (22.7) |
| BK viremia (any level) within first post-transplant year (n; %) | 45 (6) |

**Supplementary 6**: Cox regression analyses of 1-year clinical variables associated with patient mortality, graft loss (graft failure or death with functioning graft), and death-censored graft loss. Hazard ratios and 95% confidence intervals are provided for each variable included within the model. Tacrolimus IPV is included as a categorical variable.

|  | **PATIENT MORTALITY** | | **GRAFT LOSS** | | **DEATH-CENSORED GRAFT LOSS** | |
| --- | --- | --- | --- | --- | --- | --- |
| Harrell’s C-Statistic (concordance probability) and 95% confidence interval | 0.79 (0.75-0.83) | | 0.75 (0.71-0.79) | |  | |
| ***Clinical Variable*** | ***Hazard ratio*** | ***95% confidence interval*** | ***Hazard ratio*** | ***95% confidence interval*** | ***Hazard ratio*** | ***95% confidence interval*** |
| Recipient age at transplantation | **1.071** | **1.050 to 1.093** | **1.038** | **1.023 to 1.053** | 1.006 | 0.9848 to 1.028 |
| Female Sex [reference = male] | 0.9257 | 0.5591 to 1.502 | 1.274 | 0.8529 to 1.881 | **2.256** | **1.224 to 4.132** |
| Ethnicity [black; reference = white] | 0.8816 | 0.5063 to 1.493 | 0.7471 | 0.4675 to 1.166 | 0.7069 | 0.3220 to 1.459 |
| Ethnicity [Asian; reference = white] | 1.217 | 0.7171 to 2.032 | 0.9094 | 0.5893 to 1.384 | 0.8738 | 0.4495 to 1.656 |
| Donor Age | **1.021** | **1.004 to 1.040** | 1.006 | 0.9922 to 1.020 | 0.9827 | 0.9624 to 1.004 |
| CRF at at baseline | 1.003 | 0.9956 to 1.010 | 0.9999 | 0.9939 to 1.006 | 0.9944 | 0.9849 to 1.003 |
| HLA MM1 [reference = HLA MM 0] | 0.3306 | 0.04744 to 1.483 | 0.5964 | 0.1298 to 2.081 | 1.231 | 0.1591 to 7.633 |
| HLA MM 2 [reference = HLA MM 0] | 0.5738 | 0.2309 to 1.628 | 1.002 | 0.4768 to 2.366 | 1.157 | 0.3596 to 5.156 |
| HLA MM 3 [reference = HLA MM 0] | 0.6611 | 0.2797 to 1.828 | 0.7381 | 0.3556 to 1.733 | 0.9470 | 0.3065 to 4.146 |
| HLA MM 4 [reference = HLA MM 0] | 0.5205 | 0.2141 to 1.463 | 0.5996 | 0.2739 to 1.457 | 0.9295 | 0.2722 to 4.281 |
| HLA MM 5 [reference = HLA MM 0] | 0.7788 | 0.2857 to 2.338 | 0.7837 | 0.3244 to 2.020 | 0.6455 | 0.1148 to 3.621 |
| HLA MM 6 [reference = HLA MM 0] | 0.8229 | 0.2279 to 2.854 | 1.183 | 0.4001 to 3.421 | 1.569 | 0.1953 to 10.18 |
| Creatinine at 1 year | 1.002 | 0.9997 to 1.005 | **1.007** | **1.005 to 1.008** | **1.009** | **1.007 to 1.011** |
| Proteinuria at 1 year | 1.002 | 0.9998 to 1.003 | **1.004** | **1.002 to 1.005** | **1.004** | **1.002 to 1.005** |
| Rejection episode at any time in 1^st^ year  [reference = no rejection] | 2.006 | 0.9915 to 3.793 | **2.717** | **1.612 to 4.412** | **4.282** | **2.053 to 8.479** |
| CMV viremia >3000 copies/ml at 1 year  [Reference CMV negative] | 1.118 | 0.6861 to 1.776 | 0.9506 | 0.6293 to 1.406 | 0.6463 | 0.3036 to 1.266 |
| BK viremia any level at 1 year  [Reference BKV negative] | 1.497 | 0.6002 to 3.195 | 1.529 | 0.8030 to 2.680 | 1.543 | 0.5775 to 3.444 |
| Tacrolimus IPV at 1 year >50% [reference = IPV <50%] | 1.071 | 0.5221 to 2.004 | **1.836** | **1.098 to 2.932** | 1.843 | 0.8288 to 3.723 |

**Supplementary 7:** Cox regression analyses of the association of creatinine and proteinuria with death-censored graft loss. Models were created with (Model 2) and without (Model 1) the inclusion of tacrolimus IPV. Hazard ratios and 95% confidence intervals are provided for each variable included within the model. Tacrolimus IPV is included as a continuous variable.

| ***Clinical Variable*** | ***Hazard Ratio*** | ***95% confidence interval*** |
| --- | --- | --- |
| **Model 1. Harrel’s C-statistic 0.816 (95% CI 0.767-0.865)** | | |
| Creatinine at 1 year | 1.007 | 1.005 to 1.008 |
| Proteinuria at 1 year | 1.003 | 1.002 to 1.004 |
| **Model 2. Harrel’s C-statistic 0.819 (95% CI 0.767-0.871)** | | |
| Creatinine at 1 year | 1.007 | 1.005 to 1.009 |
| Proteinuria at 1 year | 1.003 | 1.002 to 1.004 |
| Tacrolimus IPV at 1 year | 1.012 | 1.003 to 1.020 |

**Supplementary 8**: Summary of previous studies investigating causes of graft failure at 1-5 years

| **References** | **Study details** | **Key Findings** |
| --- | --- | --- |
| *Betjes MGH et al. (2022)* | Netherlands; Retrospective cohort study; 737 kidney transplant recipients (1995-2005) followed until 2021, divided into age groups (18-39, 40-55, >55 years); Biopsy data analyzed | Rejection (dominant cause); TCMR decreases after 6 years, ABMR increases (1.1%/year), strongly linked to pre-transplant DSA  Death with functioning graft in elderly (43% of graft loss). |
| *Matas et al. (2002)* | University of Minnesota; Retrospective cohort study; 1467 primary kidney transplants (1990-1999); Graft loss analyzed at 1 year, 1-5 years, and >5 years post-transplant; Compared to 1980s data. | 1^st^ year - graft loss from thrombosis and death with functioning graft. Later loss (1-5 year and beyond) due to chronic rejection, non-compliance and death with functioning graft.  Compared to 1980s, Acute rejection rates decreased, thrombosis and noncompliance remained unchanged. |
| *Gaston RS et al. (2018)* | Multiple centers in the USA; 3678 kidney transplant recipients ; Mean 4.7 years follow up; Prospective cohort analysis assessing early versus late events affecting death-censored graft failure | Acute rejection was the most common early cause of graft failure (40%)  Late graft failure is often linked to new-onset allograft dysfunction, challenging the notion that early events overwhelmingly dictate long-term outcomes. |
| *Redondo-Pachón et al. (2023)* | Hospital del Mar, Barcelona, Spain; Retrospective cohort study; 1522 kidney transplants (1979-2019); Analyzed causes of graft loss in two periods (1979-1999 and 2000-2019). | Chronic allograft dysfunction was the leading cause of late graft loss in both periods with ABMR and IF/TA as main histological findings in graft failure.  Vascular thrombosis replaced TCMR as the main cause of early graft loss (2000-2019). |
| *Parajuli S et al. (2019)* | University of Wisconsin; Retrospective cohort study; 329 kidney transplant recipients (2006-2016) with graft failure within 1 year of biopsy; Analyzed histopathological findings in graft biopsies. | IFTA (53%), acute rejection (43%), and transplant glomerulopathy (33%) were the most common biopsy findings. AR was the most frequent cause of early (<6 years) graft failure, while TG was most common in late (>6 years) graft failure. The most common time for graft failure was within two years post-transplant (36%). |
| *Ponticelli et al. (2002)* | Maggiore Milano, Milan, Italy; Retrospective cohort study; 864 adult renal transplant recipients (1983-2000) with functioning grafts at 1 year; followed for up to 13 years; Multivariate analysis of risk factors for late graft failure | Chronic allograft nephropathy was the leading cause of late graft failure (49.2%), followed by death (35.5%). At multivariate analysis, elevated plasma creatinine and LDL at 1 year, older recipient age, and delayed graft function were significantly associated with late graft failure. |
